# Trouble Kills

**Published:** 1857-07

**Authors:** 


### TROUBLE KILLS.

THE secret sorrow of the mind, a sorrow which must be kept; how it wilts away the whole man, himself all unconscious meanwhile of its murderous effect! He cannot feel that he is approaching death, because he is sensible of no pain; in fact, he has no feeling, but an indescribable sensation perceived about the physical heart. Lord Raglan, commander-in-chief of the British army before Sebastopol, the bosom friend of the Duke of Wellington for forty years; of whom partial friends have often said, "his character seemed without a flaw," such a man died, figuratively, of a broken heart. In a moment, almost, trouble came like a whirlwind, avalanche followed avalanche, in such quick succession, that no time was left for the torn spirit to rise above its wounds. The British government, quailing before popular clamor, left the brave old man to bear the brunt alone, because it could not afford to recall him, and yet, had not the courage to sustain him. While the tone of official communications deprived him of his sleep, weighed heavily upon him, and broke his gallant spirit, the failure at the Redan closely followed. On reaching head quarters, a letter was in waiting, which announced the death of the last surviving member of a large family of brothers and sisters; the next day, the death of a general, his old companion in arms. Next came the news, that the gallant son of Lord Lyons was sinking under his wounds. These things, coming so rapidly one after another, in the course of a few hours, as it were, caused such a change in his appearance, all unknown to himself however, that his physician had to request him to take to his bed, and within forty-eight hours, he—died, without supposing himself to be in any danger whatever.

Within a year, a worthy lady in Ohio, sickened, in consequence of some wholly groundless rumors affecting her character, in the community into which she had recently moved. She knew they were groundless, and knew the motives of the miserable wretches who originated them; but her delicate and sensitive spirit shrunk before the shock, retreated within itself, and all torn and bleeding she died!

Within a few months, a most excellent clergyman found the feelings of his people so generally against him, that he resigned his office. The resignation was accepted; but all under such circumstances, that it was really a dismissal, and that, too, for causes which ought to have made every member of the community stand up to him like a man. Conscious of his integrity, and feeling that he had been badly dealt with—his sensibilities received a shock, which carried him to a premature grave in a few days.

"You are worse than you should be from the fever you have. Is your mind at ease?" said a quick-sighted physician, to a sleepless, wasting patient. "No, it is not," was the frank reply and the last recorded words of Oliver Goldsmith, whose *Vicar of Wakefield* and *The Deserted Village* will only die with the English language. Died at the age of forty six, of a malady of the mind, from blasted hopes and unkind speeches of the world around him! He was a man whose heart was large enough and kind enough to have made a whole world happy, whose troubles arose from his humanity; yet the base things said of him, so undeserved, so malignant, and untrue, "broke his heart."

In view of these facts, let parents early impress on the minds of children—It is not what they are charged with, but what they are guilty of, that should occasion trouble or remorse; that a carping world should not blanch the cheek or break the spirit, so long as there is conscious rectitude within.

And let all learn, what the commonest humanity dictates, to speak no word, write no line, do no deed, which would wound the feelings of any human creature, unless under a sense of duty, and even then, let it be wisely and long considered.

---

TYRANNICAL.—Don't talk about the despotism of Mayor Wood or the Great Mogul. A more inflexible tyranny prevails about our very hearth stones, the tyranny of the servant girls and the milliners of New York. If our abolition friends have any surplus of philanthropy, they would do well to expend it in "*ameliorating the condition*" of their own wives.

